# PROGRAM PLANNING STRATEGIES AMONG OLDER ADULTS PARTICIPATING IN THREE PROGRAMS PROMOTING AGING-IN-COMMUNITY

**DOI:** 10.1093/geroni/igz038.1521

**Published:** 2019-11-08

**Authors:** Su-I Hou

**Affiliations:** The University of Central Florida, Orlando, Florida, United States

## Abstract

This study examines program planning strategies among older adults participating in programs promoting aging-in-community (AIC) programs. Older adults from three programs were recruited (n=290): a university-based lifelong learning program (LLP; n=110), a county neighborhood lunch program (NLP; n=84), and a village program (n=96). Mean age was 72.4 (SD=8.68) years and 78% female. Findings showed NLP participants were more likely to obtain health information from TV (p=.030), friends and neighbors (p=.016), family members (p<.001), or mailed advertisement (p<.001); while less likely to obtain health information online (p<.001). Village members preferred afternoon while NLP participants preferred morning programs (p=.025). Most desired frequency was weekly (45%) and delivered in small group modes (68%). NLP participants were more likely to report self as risk takers (29% vs. 17%) or old tradition (23% vs. 3-8%) towards new technology adoption (p<.001). Results have implications on tailored program planning for older adults in different AIC programs.

